# Fast breakthrough of resistant cytomegalovirus during secondary letermovir prophylaxis in a hematopoietic stem cell transplant recipient

**DOI:** 10.1186/s12879-019-4016-1

**Published:** 2019-05-08

**Authors:** Susanne Jung, Manuela Michel, Thomas Stamminger, Detlef Michel

**Affiliations:** 1 Diakonissenkrankenhaus und Paulinenhilfe gGmbH, Diakonie-Klinikum Stuttgart, Rosenbergstraße 38, 70176 Stuttgart, Germany; 2 Institut für Virologie, Universitätsklinikum Ulm, Albert-Einstein-Allee 11, 89081 Ulm, Germany

**Keywords:** Antiviral resistance, Cytomegalovirus, Letermovir, Genotyping, Allogenic hematopoietic-cell transplantation, Acute myeloid leukemia

## Abstract

**Background:**

The compound letermovir (LMV) has recently been approved for the prophylaxis of cytomegalovirus (CMV) infection and disease in adult CMV seropositive recipients of an allogeneic hematopoietic stem cell transplant. LMV inhibits CMV replication by binding to the viral terminase complex. However, first cases of clinical LMV resistance have been occurred. Here we report a fast breakthrough of resistant cytomegalovirus during secondary LMV prophylaxis in a hematopoietic-cell transplant recipient.

**Case presentation:**

A 44-year-old male patient with acute myeloid leukemia (AML) experienced a CMV-reactivation within the first 4 weeks of allogeneic hematopoietic-cell transplantation. Administration of LMV was initiated at day + 34. Due to increasing viral loads, LMV treatment was discontinued after 8 days. The patient was then administered with valganciclovir (valGCV) until viral DNA was undetectable. Due to neutropenia, valGCV treatment was switched to LMV secondary prophylaxis. For 4 weeks, the patient maintain virologic suppression. Then, CMV viral loads increased with a fast kinetic. Genotypic testing of the viral polymerase UL54, the kinase UL97 as well as the viral terminase UL56 and UL89 revealed the mutation C325Y in UL56, which is associated with the high level LMV resistance.

**Conclusion:**

It is known that Letermovir is approved for prophylactic purposes. However, it may be used for some patients with CMV infection who either have failed prior therapies or are unable to tolerate other anti-CMV compounds. Particularly, the administration of LMV should be avoided in patients with detectable viral loads. When this is not possible, viral load must be routinely monitored along with UL56 genotyping. Furthermore, LMV administration at high virus loads may foster the rapid selection of resistant CMV mutants.

## Background

Cytomegalovirus (CMV) is still one of the reasons for causing severe complications after allogeneic hematopoietic-cell transplantation [1, 2]. Ganciclovir (GCV) and valganciclovir (valGCV) are routinely used for treating CMV infection in solid-organ transplantation. However, after hematopoietic-cell transplantation, GCV and valGCV are avoided due to the possibility of myelosuppression [3–5]. All traditional anti-CMV agents like GCV, foscarnet, and cidofovir target the viral DNA polymerase [6]. In contrast, the compound letermovir (LMV) inhibits CMV replication by binding to components of the viral terminase complex and therefore offers a different mode of action [7–9]. In a recently published phase III study, CMV-seropositive transplant recipients received LMV at either a dose of 240 mg per day for patients taking cyclosporine A or 480 mg per day for patients without cyclosporine co-medication (https://www.accessdata.fda.gov/drugsatfda_docs/labe/2017/209939orig1s000,209940orig1s000lbl.pdf) [10]. The lack of cross-resistance with other anti-CMV compounds, the absence of myelosuppression as well as the positive results from the phase III study led to the recent approval of letermovir for the prophylaxis of CMV infection in CMV-seropositive adult hematopoietic-cell transplant recipients.

Genotypic resistance testing was performed directly from patient specimens. Therefore, fragments of 2100 bp of the open reading frame (ORF) UL56 and 800 bp of the ORF UL89 were amplified by polymerase chain reaction followed by Sanger sequencing. The sequencing analyses of UL56 and UL89 allow detection of all known mutations conferring LMV resistance. Genotyping of the viral polymerase UL54 and the UL97 kinase were performed as previously described [11].

## Case presentation

**Fig. 1:**
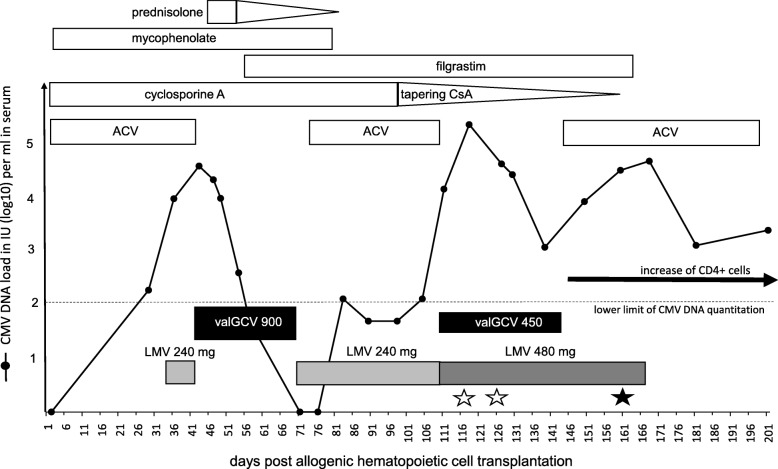
Time course of antiviral treatments and events. The CMV DNA loads (detection limit of 50 international units per ml (IU/ml)) were determined on serum samples. CMV DNA loads below the lower limit of quantization (125 IU/ml) are not to scale. The durations and the daily dosages of anti-CMV treatments with valganciclovir (450 mg or 900 mg twice a day) and letermovir are shown as black and grey boxes, respectively. Acyclovir (400 mg twice a day) was administered, when valGCV was paused. First detection of mutation C325Y (cytosine at amino acid 325 to tyrosine) in the specimens is depicted by a black star. The retrospective analysis revealed that the mutation was already present in earlier specimens shown by white stars. CsA, cyclosporine A; LMV, letermovir; valGCV, valganciclovir. ACV, acyclovir

The 44-year-old male acute myeloid leukemia (AML) patient received an unmanipulated graft from an unrelated donor (CMV D−/R+) after conditioning with the FLAMSA protocol. The patient received acyclovir (ACV, 400 mg twice per day) continuously, except between days + 43 to + 70 and day + 110 to + 145 (summarized in Fig. 1). For maintenance of immunosuppression, the patient received cyclosporine A per os (measured blood concentrations 180–220 μg/L), mycophenolate (360 mg twice daily), and prednisolone.

It was planned to start LMV prophylaxis directly after the transplantation. However, due to a delay in delivery, administration of LMV could only be initiated at day + 34, under the assumption that CMV viral load was still below detection limit (50 IU/ml in serum). The compound was given at 240 mg once per day per os, along with cyclosporine. In retrospect, it turned out that the virus DNA load at the last check on day + 28 was 190 IU/ml in serum. Over the next 8 days, increasing CMV loads were measured up to 39.600 IU/ml. Therefore, letermovir treatment was discontinued and the patient was switched to valganciclovir (valGCV, 900 mg twice per day) at day + 42. Treatment was maintained for 4 weeks until CMV DNA was negative. At this time, the patient suffered from an intestinal graft-versus-host disease (GvHD) and a mucositis. Therefore, prednisolone was administered at day + 46 for 7 days with 10 mg and then was reduced to 1 mg until discontinuation at day + 82.

As neutropenia occurred during valGCV therapy, stimulation with G-CSF was necessary. After discontinuation of valGCV, neutropenia was resolved and LMV secondary prophylaxis was started at day + 70 with 240 mg once per day. At this time point CMV DNA was not detectable. At day + 80, mycophenolate was discontinued. For 4 weeks, CMV DNA remained undetectable or at the limit of quantitation of 125 IU/ml. At day + 97, tapering of cyclosporine A was initiated. However, several days later, the patient failed to maintain virologic suppression. CMV viral loads rapidly increased to 236.400 IU/ml in serum samples, between days + 104 and + 110. In order to avoid neutropenia, valGCV treatment with a reduced dosage of 450 mg twice per day was initiated at day + 110. Concomitantly, LMV dosage was increased to 480 mg per day. During the next 4 weeks, CMV viral loads decreased to 1.200 IU/ml. ValGCV treatment, which meanwhile necessitated daily stimulation with G-CSF, was discontinued.

However, CMV DNA levels increased up to 33.000 IU/ml during the following 2 weeks. Therefore, genotyping of the CMV terminase UL56 as well as the other relevant genes (UL97 kinase, viral polymerase UL54, and UL89) was initiated. Thereby, a mutation corresponding to amino acid 325 (C325Y, cytosine at amino acid 325 to tyrosine) of UL56 was detected (see Fig. 1). This mutation is associated with a high resistance to LMV in vitro [12]. Therefore, LMV was discontinued at day + 167.

No further mutation was detected. Retrospective analysis revealed that the UL56 mutation C325Y was already present at day + 117, within 6 weeks after the start of the second letermovir administration. Unfortunately, no other patient specimens were available in order to further elucidate the time point of emergence of the mutation.

Since then CMV DNA loads remained at a low level of 1300 to 2500 IU/ml. Due to the lack of clinical symptoms and increasing CD4 T-cells since day + 145, no further anti-CMV therapy was carried out.

Until today, the patient is clinically stable and participates in a professional reintegration.

## Discussion and conclusions

Recent clinical studies indicated that letermovir might be an important addition to the current strategies for prevention of active CMV infection and disease after allogeneic hematopoietic-cell transplantation and it may be useful for salvage therapies in solid organ recipients [13, 14]. However, since the approval of the compound at the end of 2017, several patients have been reported who developed genotypically confirmed resistance to LMV while on therapy [15–18]. Thereby, LMV resistance emerged both in solid organ transplant and in hematopoietic stem cell transplant recipients. In nearly all cases which have been reported, LMV was used for salvage treatment, with considerable viral loads and for longer exposure times. In one case a breakthrough of CMV disease in a HSCT recipient occurred after more than 4 months of letermovir prophylaxis [18]. Contrary to the later, in the present case, we observed a rapid breakthrough of a resistant CMV upon secondary prophylaxis with LMV. Our patient has been re-challenged with the compound after a first short time exposure of 8 days. Thus, the question arose whether the selection of LMV-resistant viruses has already occurred during the first period of administration, because at that time virus loads were very high. On the other hand, re-administration of the drug resulted in successful suppression of virus replication for at least 30 days. Then, however, the resistant CMV subpopulation apparently prevailed. Unfortunately, due to the lack of appropriate patient specimens the question concerning the exact time point of emergence of LMV resistance cannot be answered unequivocally. Additional studies are needed to decide whether the re-exposure to letermovir during repeated periods of CMV reactivation poses a particular risk for the development of antiviral drug resistance.

Another question is, whether the dosage of LMV was too low for virus suppression at the time when cyclosporine was tapered. In a phase II prophylaxis trial, a single case of breakthrough CMV viremia on LMV prophylactic treatment had been published. Thereby, the UL56 mutation V236 M emerged on a daily dose of 60 mg suggesting that low-dose prophylaxis may confer letermovir resistance [19]. However, although cyclosporin is known to alter LMV pharmacokinetics, a dosage of 240 mg letermovir even in the absence of CsA has been reported to be sufficient for complete suppression of viremia [19, 20].

We cannot exclude that individual reasons in this specific patient (e.g. compliance issues, nausea or atypical metabolism) led to low drug levels since no pharmacokinetic monitoring of LMV is available. Nonetheless, it is presently unclear whether higher doses of the drug would be more effective or safe during prolonged use since a recent study reported two patients who developed genotypically confirmed resistance to LMV while on therapy with 720 mg and 960 mg daily, respectively [16].

In vitro studies with serial viral passage in the presence of the compound have been associated with relatively rapid selection of several different UL56 mutations, particularly within codons 25 and 231 to 369 [21, 22]. Thereby, the different mutations led to diverse levels of resistance, ranging from a 5-fold increase in EC50 for V231L to > 5000-fold for C325Y [21, 22]. Remarkably, in all reports published after the approval of letermovir, the amino acid 325 in UL56 was exclusively affected [15–18]. In our case the mutation is also located at this codon. This suggests that amino acid 325 of UL56 may represent a hot spot for the occurrence of resistance upon clinical treatment. The presently available data suggest the necessity for fast genotyping for early detection of relevant mutations before treatment failure is evident.

According to the currently available data, letermovir should, if possible, only be used within the scope of the approval. Administration in patients at high risk with active CMV infections and considerable virus loads requires caution and close clinical and virological monitoring concerning the emergence of drug-resistant virus variants. Furthermore, LMV administration at high virus loads combined with (short time) drug exposures may foster the rapid selection of resistant CMV variants.

## Abbreviations

allo-HCT: Hematopoietic-cell transplantation
AML: Acute myeloid leukemia
CMV: Human cytomegalovirus
LMV: Letermovir
PCR: Polymerase chain reaction
UL54: Unique long open reading frame 54
UL56: Unique long open reading frame 56
UL89: Unique long open reading frame 89
UL97: Unique long open reading frame 97
